# Sodium Supplementation Algorithm to Promote Growth in Infants Who Are Preterm: Randomized Clinical Trial

**DOI:** 10.1542/pedsos.2025-001089

**Published:** 2026-02-20

**Authors:** Brianna M. Liberio, Gregory M. Sokol, Hannah Rakow, Kelly Mosesso, Valerie Hole, William W. Wong, Shaji K. Chacko, Jeffrey L. Segar

**Affiliations:** 1 Department of Pediatrics, Indiana University School of Medicine, Indianapolis, Indiana; 2 Department of Biostatistics and Health Data Science, Indiana University School of Medicine, Indianapolis, Indiana; 3 Department of Physiology, Medical College of Wisconsin, Milwaukee, Wisconsin; 4 USDA/ARS Children’s Nutrition Research Center, Department of Pediatrics, Baylor College of Medicine, Houston, Texas; 5 Department of Pediatrics, Medical College of Wisconsin, Milwaukee, Wisconsin

## Abstract

**OBJECTIVE:**

Evaluate the effectiveness of an algorithm using urine sodium (Na) concentration to guide dietary Na supplementation compared with standard care to promote growth in infants who are preterm.

**METHODS:**

A randomized trial was conducted in 4 neonatal units in Indianapolis, Indiana, involving infants between 25^0/7^ to 29^6/7^ weeks gestation with birthweight > 500 g. Study arms consisted of standard care or use of algorithm beginning at 14 to 17 days postnatal age. The primary outcomes were somatic growth (weight, length, and head circumference) measured by change in Z-score between aged 2 weeks and 36 weeks postmenstrual age (PMA). Secondary outcomes included total body water and body composition.

**RESULTS:**

Of 260 eligible infants, 90 were consented and randomized, with 45 allocated to each arm and 43 in each arm completing the study. Thirty-eight (88%) infants randomized to the study algorithm had a low urine Na that qualified for Na supplementation (4 mEq/kg/d). Infants assigned the algorithm received more Na (5.75 ± 0.96 mEq/kg/d) than infants assigned the standard care (3.62 ± 1.25 mEq/kg/d); caloric intake did not differ. Change in weight Z-score from study entry until 36 weeks PMA was not statistically different between groups (0.16 ± 0.41 vs 0.04 ± 0.63, algorithm vs control; *P* = .16). Infants assigned the algorithm displayed a significantly more rapid increase in weight between weeks 0 to 7 of the study than infants assigned the standard care. Total body water and fat-free mass did not differ at 32 weeks PMA.

**CONCLUSION:**

Na supplementation guided by urine Na concentration did not significantly improve weight gain in infants 25^0/7^ to 29^6/7^ weeks gestation at 36 weeks PMA.

## Introduction

Despite aggressive parenteral and enteral nutrition, suboptimal extrauterine growth remains a significant concern in infants who are preterm.^1^ Strong associations have been identified between in-hospital growth and impaired short- and long-term neurodevelopment.^2–4^ It is imperative that other nutritional strategies, including a focus on micronutrients, be adapted to address this major issue.

For almost a century, the importance of adequate dietary sodium (Na) intake in supporting somatic growth has been recognized.^5^ In young animals, an Na-deficient diet impairs weight and length gain through decreased energy efficiency and increased basal metabolic rate, whereas salt supplementation to Na-depleted animals restores growth.^5–9^ Infants who are preterm are at risk for Na depletion because of large, underappreciated urine Na (U_Na_) losses coupled with the low Na content of human milk and infant formula.^10,11^ We previously demonstrated that infants who are preterm born at gestational ages 22 to 29 weeks have U_Na_ losses up to 5 to 8 mEq/kg/d over the first 4 to 8 weeks after birth, often exceeding Na intake.^10,12^ Sodium supplementation above that provided in the standard diet optimizes weight gain in infants who are preterm.^13–16^ Because these studies provided Na supplementation without an assessment of Na deficiency, Segar et al proposed a tailored approach, applying an algorithm using U_Na_ concentration to screen for Na deficiency and guide Na supplementation.^10^ In this pre- and postimplementation study, the postimplementation cohort demonstrated a greater increase in weight Z-score between 2 and 8 weeks after birth compared with the preimplementation cohort despite similar caloric and protein intakes. A recent, expanded retrospective study of infants cared for before and after implementation of this algorithm found similar results.^17^ These reports were not randomized trials, and concern that other clinical practices may have impacted growth exists.

We therefore performed a randomized trial to assess the impact of this study algorithm (SA) to guide dietary Na supplementation compared with standard care (SC) on somatic growth in infants who are preterm. We hypothesized that the SA would result in improved somatic growth compared with SC. We additionally hypothesized that use of the SA would result in no difference in total body water (TBW) or body composition at 32 weeks postmenstrual age (PMA) or common morbidities associated with preterm birth.

## Methods

### Trial Design

This prospective, randomized trial was conducted from July 1, 2019, to August 15, 2023, in 3 level III neonatal intensive care units (NICUs) and 1 level IV NICU in Indianapolis, Indiana, and surrounding metropolitan area. The study was approved by the Indiana University Institutional Review Board (#1902815407), followed the Consolidated Standards of Reporting Trials guidelines, and was registered at ClinicalTrials.gov (NCT03889197).

### Study Participants

Patients were eligible if born between 25^0/7^ and 29^6/7^ weeks of gestational age (GA) with a birth weight ≥500 g and admitted to a participating site within the first week after birth. Exclusion criteria were admission after first week after birth, major congenital anomalies, structural genitourinary abnormalities, intestinal ostomies, diabetes insipidus, diuretic use within 48 hours of study initiation, and kidney dysfunction (serum creatinine >1.0 mg/dL or an increase of ≥0.3 mg/dL between the 2 most recent consecutive measurements prior to study initiation).^18^

### Study Procedures

After obtaining the parent/guardian’s informed consent, computer-generated randomization of participants to either the SC or SA group occurred using REDCap (Research Electronic Data Capture) using a permutated block scheme stratified by GA at birth: 25^0/7^ to 27^6/7^ and 28^0/7^ to 29^6/7^ weeks. The randomization assignment was communicated to the medical care team who implemented the assignment. Prior to randomization, infant nutrition was per standard approaches in the NICU, typically providing 2 to 3 mEq/kg/d of Na while receiving parenteral nutrition. When tolerating enteral feedings, dietary Na was that contained in breast milk and fortifier or formula. Infants randomized to the SA arm had an initial U_Na_ measurement analyzed by the hospital clinical laboratory at 14 to 17 days postnatal age and subsequently every 2 weeks until 36 weeks PMA. Sodium supplementation was guided by the SA (Table 1). Infants in the SC arm received Na per discretion of the medical care team. A single urine collection was obtained with use of a urine collection bag and cotton balls. This approach avoids contamination with stool and decreases risk of leakage from the bag. Infants assigned to SA received Na supplementation if their U_Na_ was below the level outlined in the algorithm. A total of 4 mEq/kg/d was chosen as the amount of initial Na supplementation based on the study by Isemann et al.^13^ Infants in the SC arm received Na per discretion of the medical care team and did not have U_Na_ measured. Anthropometric measurements were obtained weekly by trained research nurses. Aside from the described study interventions, infants were cared for according to standard unit protocols, including incubator humidification to 50% until 32 weeks PMA and allowing weaning from incubator to crib when an infant was >1600 g.

#### Routes of Sodium Administration for Study Group

For infants receiving ≤80 ml/kg/d of enteral feedings, Na was added to parenteral fluids and infused over 24 hours. For infants receiving >80 ml/kg/d of enteral feedings, Na was added to the feedings (equally divided quantities with every other feed) or provided by a combination of enteral and parenteral route per the medical team preference and provided as chloride, acetate, citrate, bicarbonate, or any mixture thereof.

### Data Collection

Maternal and infant demographic variables, birth history, infant comorbidities, weights, and nutrition data were abstracted from medical records and entered into a REDCap database. Parenteral and enteral intakes of fluids, protein, lipid, calories, and Na were recorded each Monday, Wednesday, and Friday until 36 weeks PMA or hospital discharge (whichever occurred first). Sodium content of breast milk was estimated at 0.8 mEq/100 ml, whereas human milk fortified to 24 kcal/oz and 27 kcal/oz with human milk fortifier were estimated to have an Na content of 1.4 and 1.8 mEq/100 ml, respectively.^11,19^ Sodium content of formula was described by the manufacturer.

### Study Outcomes

The primary outcomes were differences in somatic growth (weight, length, and head circumference) as determined by the changes in Z-score between aged 2 weeks and 36 weeks PMA. Weight, length, and head circumference Z-scores were calculated based on GA-specific growth charts published by Fenton.^20,21^ Body composition and TBW were determined at 32 weeks PMA using the double-labeled water (DLW, ^2^H_2_^18^O) method (see Supplementary Materials for detailed methodology).^22–24^ Fat-free mass was determined using TBW and the developmentally appropriate hydration factor.^25^ Additional secondary outcomes included incidence and severity of bronchopulmonary dysplasia (BPD), retinopathy of prematurity (ROP), dysnatremias, duration of mechanical ventilation, need for supplemental oxygen, and use of diuretics. Energy expenditure at 32 weeks PMA determined by the DLW method will be reported elsewhere.

### Sample Size Determination

A sample size of 82 patients (41 per group) was determined based on the primary outcome of difference in weight gain between groups using previously published data to identify a difference in weight Z-score of 0.25 with an SD of 0.4, an alpha of 0.05, and power of 0.8 at 36 weeks PMA.^10^ This Z-score difference was chosen based on our previous findings using this algorithm.^10^ To account for potential withdrawal or loss from the study, including from methodological errors with the DLW experiments, and to maintain calculated power, we targeted recruiting 45 patients per group.

### Statistical Methods

The primary analysis (and all analyses examining outcomes by treatment) were adjusted for the stratification variable of GA. Because the primary outcome is continuous, linear regression was used to estimate the adjusted mean difference in growth between the 2 groups. Similarly, for secondary outcomes, linear regression was used for continuous outcomes and robust Poisson regression for binary or categorical outcomes to obtain adjusted relative risk estimates for the treatment effect. Where possible, data are expressed as mean ± SD. Details on weighted least squares regression modeling of body weight Z-scores and DLW experiment outcomes are provided in the Supplementary Methods and eTable 1. The blinding of participants and/or those assessing outcomes were not performed. The trial was monitored by a data safety monitoring committee, with evaluations at 25%, 50%, and 75% trial completion.

## Results

**FIGURE 1.**
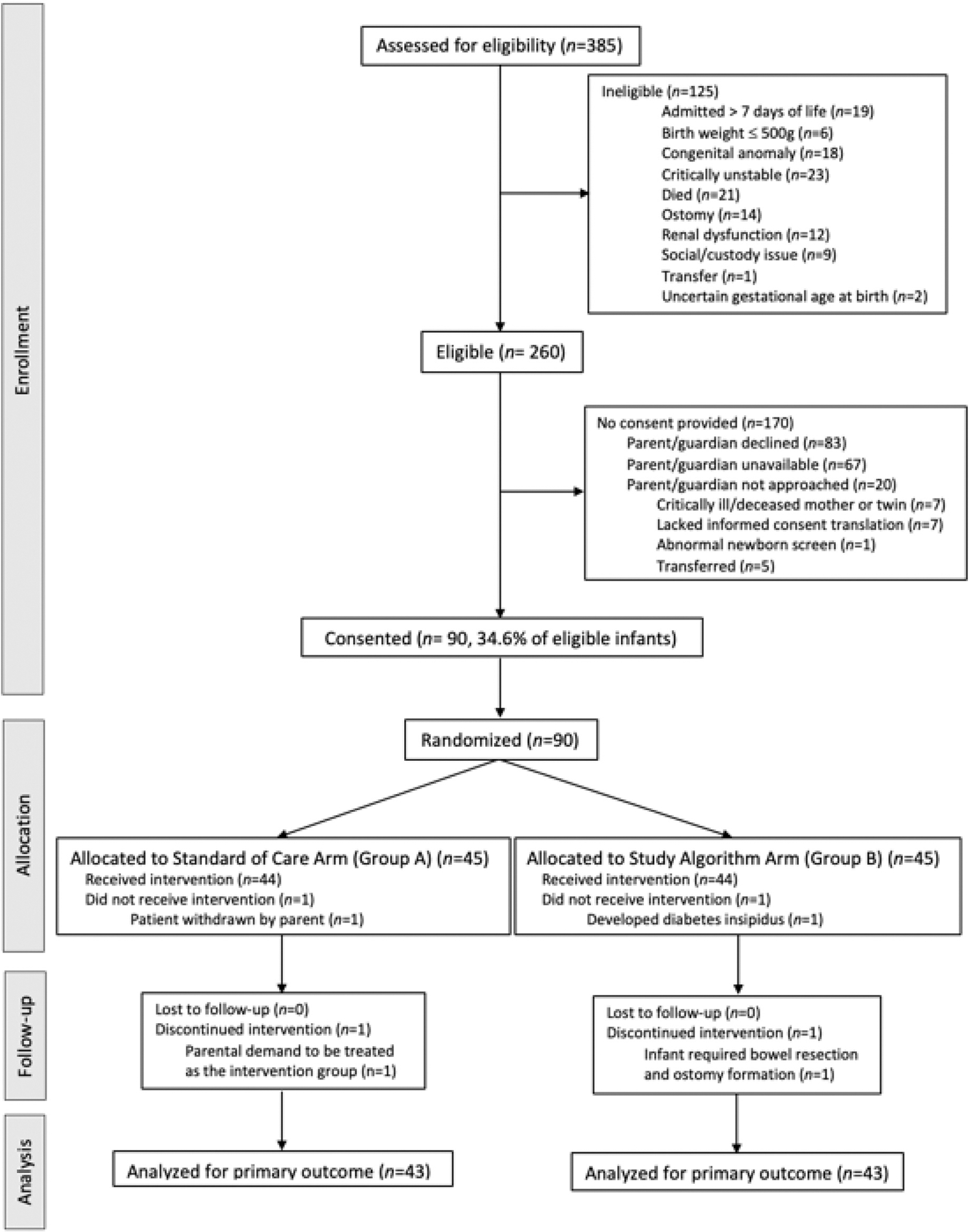
Consolidated Standards of Reporting Trials diagram providing participant identification, enrollment, and analysis.

From July 1, 2019, to July 22, 2023, 385 patients were assessed for eligibility. Of those eligible, 90/260 (34.6%) were consented and randomized. A total of 45 patients were initially allocated to each arm, with 43 patients completing the study in each arm (Figure 1). Maternal and infant characteristics are described in Table 2. There were no differences between groups in weight, length, or head circumference at birth or 2 weeks postnatal age (week 0 of study).

In the SA group, 25/43 (58%) of participants had an initial (2 weeks postnatal age) U_Na_ below the algorithm threshold and received Na supplementation. During week 3 of the study (fourth week postnatal age), 35/43 (81%) of infants assigned SA received Na supplementation, and at 6 weeks postnatal age, 38/43 (88%) received Na supplementation. During the study, 14/43 (33%) of infants assigned SA had a U_Na_ warranting further increase in Na supplementation from 4 mEq/kg/d to 6 mEq/kg/d. During the first 8 weeks of the study, average daily caloric intake did not differ between groups (SA: 120 ± 12 kcal/kg/d vs SC: 120 ± 8 kcal/kg/d), whereas average daily protein intake was higher in the SA group compared with the SC group (SA: 4.04 ± 0.41 g/kg/d vs SC: 3.83 ± 0.51 g/kg/d; *P* < .05). Over this period, SA infants received an average of 5.75 ± 0.96 mEq/kg/d of Na, whereas SC infants received 3.62 ± 1.25 mEq/kg/d (*P* < .001). Exposure to human milk or formula did not differ between groups (eTable 2 in Supplementary Materials).

No differences in change in weight, length, or head circumference Z-scores between aged 2 weeks and 36 weeks PMA were identified between groups inclusive of all infants (Table 3).

**FIGURE 2.**
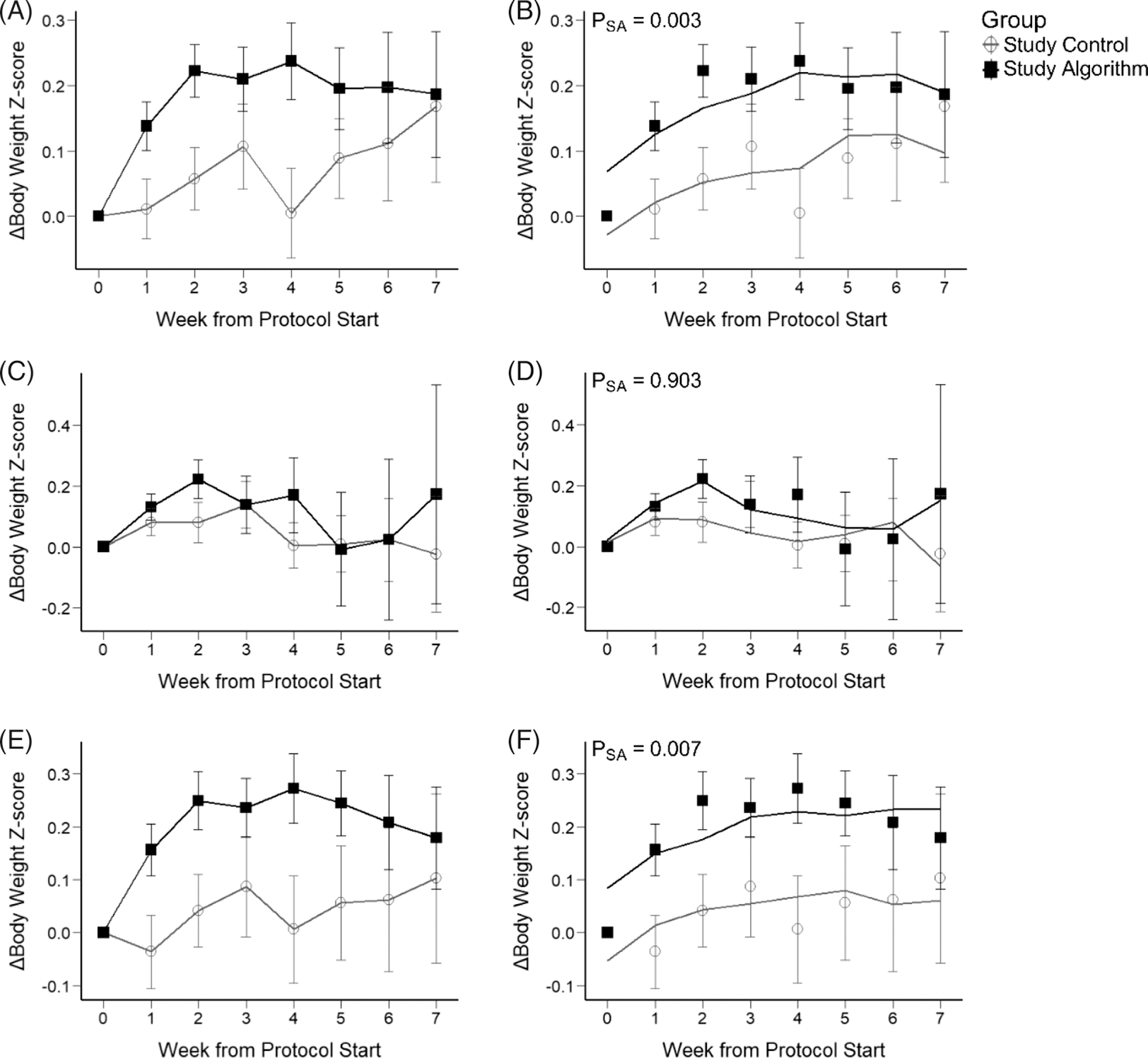
Change in weight Z-score from study entry (week 0) until week 7. Sodium supplementation, if indicated, was initiated beginning week 0. Plots include for raw data: (A) SC n = 43, SA n = 43 and linear mixed model: (B) SC n = 43, SA n = 40. Because of missing data required for linear mixed mode, 3 infants in SA group are excluded. Abbreviations: SA, study algorithm; SC, standard care.

However, when assessing growth between weeks 0 to 7 of the study, post hoc testing by weighted least squares regression modeling revealed significantly increased body weight Z-scores in infants assigned SA compared with infants assigned SC (Figure 2A and 2B). Differences in weight gain, which were most pronounced during the early to midstudy period, resulted in different growth trajectories and patterns. However, body weights became similar at the end of the study period, 36 weeks PMA (7–10 weeks postnatal age). A significant relationship between weight gain and average weekly caloric intake but not protein or Na intakes was identified (see eTable 1 in Supplementary Materials).

**FIGURE 3.**
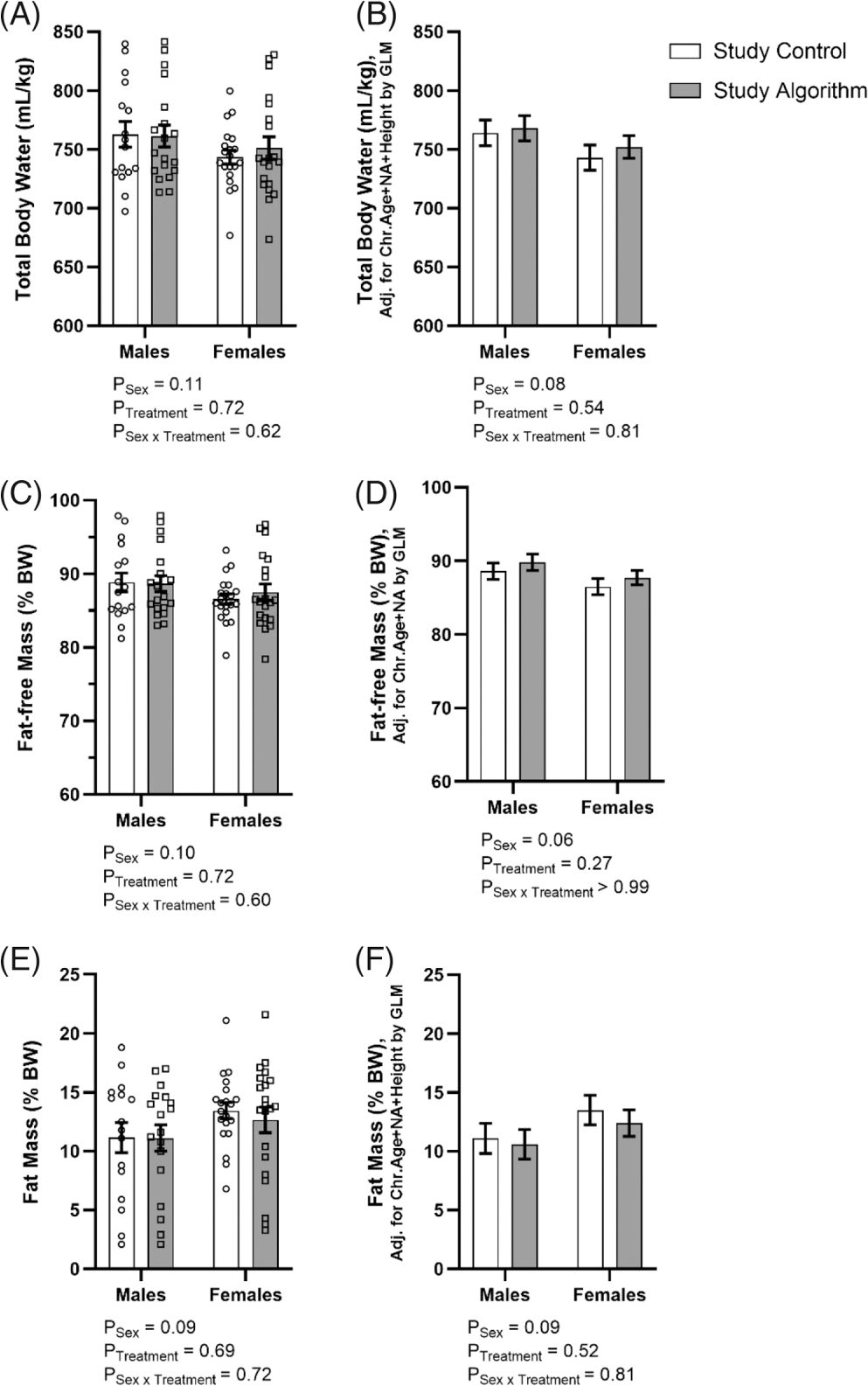
Double-labeled water studies measuring (A, B) total body water (ml/kg BW), (C, D) fat-free mass, (E, F) and fat mass in male and female infants at 32 weeks postmenstrual age. Total body water, fat-free mass, and fat mass data are unadjusted (A, C, E) and adjusted for chronological age and sodium intake (B, D, F). Male SC n = 16; male SA n = 16; female SC n = 20; female SA n = 20 (mean ± SEM, two-way analysis of variance, Šídák’s multiple comparisons test on estimated least square means). Abbreviations: BW, body weight; GLM, generalized linear model; NA, sodium intake; SA, study algorithm; SC, standard care.

DLW studies were completed at 32 weeks PMA on 83 infants; 7 infants were excluded from analysis because the percentage of isotope dilution spaces relative to body weight was over 90%, which is not physiologic. This rate of study failure is consistent with other studies in preterm infants using DLW (W. Wong, 8/12/2024, email). Data were available from 16 male SA, 19 male SC, 20 female SA, and 20 female SC infants. The percentage of TBW relative to body weight did not differ between treatment groups for boys (SC: 76.3 ± 1.1 vs SA: 76.1 ± 0.9%) and girls (SC: 74.4 ± 0.6 vs SA: 75.1 ± 1.0%). No significant effects of chronological age, sex, or average daily Na intake on %TBW were identified (Figure 3A and 3B).

Fat-free mass (FFM) did not differ between groups, with no significant effects of chronological age, sex, or average daily Na intake being identified (Figure 3C and 3D). Fat mass (body weight – FFM) also did not differ between treatment groups, with no significant effects of sex or chronological age (Figure 3E and 3F). No differences between groups were identified in incidences of dysnatremias, BPD at 36 weeks PMA, severe ROP, receipt of diuretics, or discharge with supplemental oxygen or duration of supplemental oxygen, mechanical ventilation, or hospital stay (Table 4).

## Discussion

In this trial of a clinical practice algorithm using U_Na_ to guide Na supplementation in infants born between 25^0/7^ and 29^6/7^ weeks GA, we found no significant difference between groups in the primary outcomes of change in weight, length, or head circumference Z-scores between 2 weeks postnatal age and 36 weeks PMA. Infants treated with the algorithm did, however, show a more rapid increase in weight gain over the first 7 weeks of study. No differences in total body water or body composition were identified between groups at 32 weeks PMA or in the rates of BPD, ROP, NEC, or length of hospital stay. The lack of effect on growth at 36 weeks PMA contrasts findings of previous studies that demonstrate that dietary Na supplementation of 2 to 4 mEq/kg/d above that typically prescribed infants who are preterm improves weight gain. Various methodological approaches to Na supplementation, duration of treatment, infant gestation ages, growth end points, and sample size may contribute to the differences in findings.^10,13,15–17^

The rationale for using U_Na_ concentration to identify infants who may benefit from Na supplementation is based on the observation that in response to body Na depletion, renal conservation of Na results in a low U_Na_ concentration.^26,27^ Renal tubular immaturity and developmental changes in renal Na handling confound this approach in infants who are preterm.^10,28^ Based on our previous modeling, we adapted algorithm U_Na_ concentrations that were conservative in identifying infants with Na deficiency and guiding Na supplementation. We previously reported that following adoption of the algorithm in the University of Iowa NICU, change in weight Z-scores between aged 2 and 8 weeks was significantly greater in infants treated with the algorithm 26 to 29^6/7^ weeks gestation compared with historical (0.32 ± 0.05 vs −0.01 ± 0.08; *P* < .01, n = 40–50 per group).^10^ The difference in Na intakes between groups, which was estimated using reference values for the Na content of human milk, was approximately 2 mEq/kg/d, similar to that seen in the present study. A more recent report from this same NICU involving over 300 infants 26 to 29^6/7^ weeks gestation cared for between 2012 and 2020 found a mean difference in change in weight Z-score of 0.15 (95% CI, 0.06–0.25; *P* < .005).^17^

Improved growth in infants who are preterm who receive Na supplementation has been demonstrated in several studies. Isemann et al identified infants <32 weeks GA receiving Na supplementation of 4 mEq/kg/d from postnatal day 7 to 35 had greater weight gain from birth to 6 weeks of postnatal age than controls.^13^ Ayisi et al similarly reported that very low birth weight infants fed mothers milk had significant increases in weight, length, and head circumference when supplemented with Na (3 mEq/kg/d).^14^ More recently, Amiti et al reported significantly increased weight gain and linear growth in infants 25 to 30^6/7^ weeks gestation randomized to receive Na (4 mEq/kg/d) compared with placebo, beginning once receiving enteral feeds of 100 ml/kg/d or aged 10 days.^16^ A recent retrospective study including infants <32 weeks gestation identified that infants receiving Na supplementation experienced significant increases in weight gain after starting supplementation compared with nonsupplemented infants at similar PMA.^15^

The differences in the pattern of growth between SA and SC groups are intriguing. The impact of Na supplementation on growth was most pronounced in the early intervention window, within the first 4 weeks after randomization. The pattern of “delayed growth” followed by “catch-up growth” in the SC group is similar to that seen in preclinical studies in which rodents maintained on a low Na diet demonstrate impaired growth but rapid weight gain when returned to a standard Na diet.^8,9,29,30^ We speculate that the relatively slower growth in infants assigned SC results from inadequate Na intake, although with kidney maturation, the amount of Na in the diet becomes sufficient to replenish body sodium and support growth. Differences in fluid intake, not tracked in this study, and use of diuretics may also have impacted weight changes over the duration of study.

We acknowledge limitations of using U_Na_ for assessment of Na homeostasis in infants who are preterm, although U_Na_ has emerged as a useful tool in other populations. Low U_Na_ has been correlated with poor growth in infants with intestinal ostomies and cystic fibrosis, whereas Na supplementation to achieve U_Na_ > 30 mEq/L is associated with improved weight gain.^31–34^ Based on our previous work, we identified a U_Na_ of 40 meq/L as a reasonable, conservative value at which to initiate Na supplementation.^10^ Infants born <25 weeks GA were not included in the present study, although it warrants an additional study knowing they experience a prolonged period of high U_Na_ losses.^12^ Sodium supplementation in the present study did not begin prior to 2 weeks of postnatal age, although the optimal time to begin Na supplementation to promote a positive Na balance is not known.

No differences in TBW at 32 weeks PMA were identified between groups, suggesting that the increased weight gain seen in the SA group did not result from disproportionate water accumulation. This finding was not surprising given studies in rats demonstrated that 30-fold differences in Na intake over a 5-week period resulted in significant differences in growth but not TBW or serum Na concentration values.^7^ Similarly, in adult men, 11-fold differences in Na intake over 7 days failed to produce differences in TBW or mass.^35^ These findings should be reassuring regarding concerns of increased Na intake resulting in water retention.

The incidences of comorbidities were unchanged by SA adherence and increased Na intake. These results are in accordance with previous work suggesting Na supplementation to the extent provided in the current study is not associated with increased incidence of BPD, hypertension, necrotizing enterocolitis, or hypernatremia.^15–17^ Collectively, these findings provide reassurance that Na supplementation provided in the quantities of this study does not increase risk for morbidity.

We used U_Na_ to identify infants who may be Na deficient and thus benefit from Na supplementation. However, given that the majority of infants in the SA arm received Na supplementation based on a low U_Na_, (thus presumed Na deficient), a general approach of increasing the standard amount of dietary Na that infants who are preterm receive may be appropriate. This study and others highlight the high probability that many infants who are preterm fail to receive sufficient dietary Na necessary to maintain Na homeostasis and support optimal growth. Current evidence suggests Na intakes consistent with recent recommendations of the European Society of Pediatric Gastroenterology, Hepatology and Nutrition Committee on Nutrition, including Na intakes up to 8 mEq/kg/d depending on gestational and postnatal ages, are needed.^36^ For infants failing to grow at desirable rates despite high energy and protein intakes, measurement of U_Na_ concentration to guide Na supplementation may assist with clinical management.

## Supplementary Material

Supplementary material

## Figures and Tables

**TABLE 1.** Clinical Practice Algorithm to Guide Sodium Supplementation Using Urine Sodium Concentrations

		Postnatal Age (Weeks)					

Gestational age at birth (weeks)	2	4	6	8	10	12	14
25–25^6/7^	<50	<40	<40	<40	<40	<30	<30
26–29^6/7^	<40	<40	<40	<40	<30	<30	
**Algorithm instructions:**							
1.	Values represent urine sodium concentrations (U_Na_), expressed as mEq/L, obtained every other week. Do not obtain U_Na_ within 48 hours of diuretic administration.						
2.	If U_Na_ is below threshold value, initiate sodium supplementation at 4 mEq/ kg/day above current sodium intake. Adjust weekly to account for weight gain.						
3.	For subsequent time points, if U_Na_ is below corresponding goal value at subsequent biweekly timepoints, increase sodium supplementation by 2 mEq/kg/day.						
4.	Provide supplementation if serum [Na] ≤ 132 mEq/L regardless of U_Na_ unless there is evidence of acute fluid overload (significant increase in weight). Hyponatremia in presence of high U_Na_ likely represents a condition of severe urinary sodium loss leading to hyponatremia.						
5.	Continue supplementation unless serum [Na] >144 mEq/L. a. If serum [Na] 145–146 mEq/L, reduce sodium supplement by half and consider measuring serum [Na] on subsequent day. b. If serum [Na] ≥ 147 mEq/L, discontinue sodium supplementation.						
6.	Continuation of supplementation after 38 weeks postmenstrual age will be at the discretion of the clinical care team.						

**TABLE 2.** Maternal and Infant Characteristics

	Standard Care (n = 43)	Study Algorithm (n = 43)

**Maternal Characteristics**		
Age, mean (SD), years	29.3 (6.4)	29.9 (6.1)
Race, n (%)		
Black	14 (32.6)	11 (25.6)
White	26 (60.5)	30 (69.8)
Other	2 (4.7)	2 (4.7)
Not reported	1 (2.3)	0 (0)
Ethnicity, n (%)		
Hispanic or Latino ethnic group	8 (18.6)	5 (11.6)
Non-Hispanic or Latino	34 (79.1)	38 (88.4)
Not reported	1 (2.3)	0 (0)
Maternal diabetes requiring insulin or medications, n (%)	3 (7.0)	3 (7.0)
Maternal hypertension, n (%)	21 (48.8)	19 (44.2)
Maternal chorioamnionitis	4 (9.5)	12 (27.9)^a^
**Infant Characteristics**		
Gestational age at birth, mean (SD), weeks	28.00 (1.44)	27.87 (1.36)
Singleton, n (%)	30 (69.8)	38 (88.4)^a^
Male sex, n (%)	19 (44.2)	19 (44.2)
Inborn, n (%)	40 (93.0)	41 (95.3)
Birth weight, mean (SD), kg	1.14 (0.24)	1.13 (0.28)
Birth weight Z-score, mean (SD)	0.45 (0.93)	0.24 (0.96)
Birth length, mean (SD), cm	36.05 (3.04)	36.29 (3.03)
Birth length Z-score, mean (SD)	0.04 (1.10)	−0.11 (1.63)
Birth head circumference, mean (SD), cm	25.7 (1.8)	25.2 (2.1)
Birth head circumference Z-score, mean (SD)	0.25 (0.99)	−0.16 (1.18)
Weight at 2 weeks, mean (SD), kg	1.47 (0.31)	1.43 (0.40)
Weight at 2 weeks Z-score, mean (SD)	−0.31 (0.64)	−0.28 (0.82)
Length at 2 weeks, mean (SD), cm	40.3 (2.8)	39.8 (3.0)
Length at 2 weeks Z-score, mean (SD)	−0.52 (0.84)	−0.57 (0.80)
Head circumference at 2 weeks, mean (SD), cm	27.1 (2.0)	27.9 (2.4)
Head circumference at 2 weeks Z-score, mean (SD)	−1.11 (1.04)	−0.93 (1.02)
Severely small for gestational age (< third percentile), n (%)	2 (4.7)	2 (4.7)
Received antenatal glucocorticoids, n (%)	34 (79.1)	40 (93.0)
Cesarian delivery, n (%)	34 (79.1)	31 (72.1)
Apgar score at 1 min, median (IQR)	5 (2, 6)	5 (3, 6)
Apgar score at 5 min, median (IQR)	7 (6, 8)	7 (5, 8)
Age at randomization, mean (SD), days	14.35 (1.23)	14.33 (1.23)

a *P* < 0.05.

**TABLE 3.** Somatic Growth Outcomes

	Standard Care (n = 43)			Study Algorithm (n = 43)			
Growth Parameter	Study Entry	36 Weeks PMA	Change	Study Entry	36 Weeks PMA	Change	*P* Value

Weight, mean (SD), kg	1.28 (0.28)	2.55 (0.40)	1.27 (0.42)	1.24 (0.31)	2.54 (0.37)	1.31 (0.33)	.492
Weight Z-score, mean (SD)	−0.31 (0.67)	−0.27 (0.81)	0.04 (0.63)	−0.46 (0.68)	−0.30 (0.89)	0.16 (0.41)	.162
Length, mean (SD), cm	37.94 (2.80)	44.92 (2.38)	6.98 (2.46)	38.04 (2.88)	44.78 (2.24)	6.73 (2.51)	.465
Length Z-score, mean (SD)	−0.39 (0.92)	−0.72 (0.92)	−0.34 (0.66)	−0.35 (0.78)	−0.80 (0.89)	−0.48 (0.48)	.242
Head circumference, mean (SD), cm	25.70 (1.80)	31.60 (1.72)	5.96 (1.92)	25.59 (1.88)	31.60 (1.27)	6.01 (1.52)	.972
Head circumference Z-score, mean (SD)	−1.12 (0.88)	−0.53 (1.06)	0.60 (0.88)	−1.09 (0.80)	−0.56 (0.90)	0.54 (0.57)	.956

Abbreviation: PMA, postmenstrual age.

**TABLE 4.** Secondary Outcomes

	Standard Care (n = 43)	Study Algorithm (n = 43)	*P* Value

Hypernatremia, n (%)	5 (11.6)	8 (18.6)	.366
Hyponatremia, n (%)	10 (23.3)	10 (23.3)	1.000
Received any diuretic therapy, n (%)	7 (16.3)	11 (25.6)	.289
Duration of invasive mechanical ventilation, median (IQR), days	1.00 (0.50, 3.50)	1.00 (0.00, 9.00)	.940
Duration of supplemental oxygen, median (IQR), days	37.00 (18.50, 69.00)	42.00 (14.50, 79.00)	.704
Discharged on supplemental oxygen, n (%)	7 (16.3)	13 (30.2)	.126
Bronchopulmonary dysplasia, n (%)			.738
None	16 (37.2)	15 (34.9)	
Grade 1	11 (25.6)	10 (23.3)	
Grade 2	12 (27.9)	16 (37.2)	
Grade 3	4 (9.3)	2 (4.7)	
Retinopathy of prematurity stage ≥ 3 or treatment received, n (%)	4 (9.3)	2 (4.7)	.676
Length of hospitalization, median (IQR), days	80.00 (59.00, 97.50)	86.00 (68.00, 98.50)	.412

## WHAT IS KNOWN ON THIS SUBJECT:

Preterm infants are at risk of sodium depletion because of large and often unrecognized urine sodium losses. Sodium supplementation above that in formula or fortified breast milk helps promote growth.

## WHAT THIS STUDY ADDS:

Using urine sodium concentration to guide sodium supplementation in preterm infants 25^0/7^ to 29^6/7^ weeks gestational age may promote more rapid weight gain.

## Abbreviations

BPD: bronchopulmonary dysplasia
DLW: double-labeled water
GA: gestational age
Na: sodium
NICU: neonatal intensive care unit
PMA: postmenstrual age
ROP: retinopathy of prematurity
SA: study algorithm
SC: standard care
TBW: total body water
U_Na_: urine sodium
