# Supplementary material for: Sodium Supplementation Algorithm to Promote Growth in Infants Who Are Preterm: Randomized Clinical Trial
Source: Pediatr Open Sci. Author manuscript; Available in PMC 2026 Jul 15. (PMC13367914; doi:10.1542/pedsos.2025-001089)
Supplement: Supplementary material [file NIHMS2185970-supplement-Supplementary_material.pdf]

Supplemental material (eMethods):

Total Body Water and Body Composition Measurements

Total body wate(TBW) in the premature infants was measured using the oxygen-18 (^{18}O) isotope dilution method. The isotope dilution method is considered a reference method for body water measurements in free-living subject.[1, 2] Briefly, the method involves enrichment of body water with the stable (natural and nonradioactive) isotope of ^{18}O. For each measurement, one baseline (pre-dose) urine sample was collected from each infant. The participant then received via a nasogastric feeding tube, 1.5 g/kg of a premixed cocktail containing 0.138 g of 100% ^{18}O per kg body weight. Sterility of the dose was ensured through microfiltration at the time the isotope water was compounded by the manufacturer. The dose was flushed through the nasogastric feeding tube with 2 ml of sterile water. Post-dose urine samples were collected between 6-24 h after dosing and again 2 and 5 days after dosing. The exact date and time of dosing and sample collection were recorded. The urine samples were transferred in duplicate to pre-labeled o-ring cryovials and stored in a freezer. One set of frozen samples from each study was shipped to the Gas-Isotope-Ratio Mass Spectrometry Laboratory at the USDA/ARS Children's Nutrition Research Center for stable oxygen isotope ratio measurements by continuous-flow gas-isotope-ratio mass spectrometry. For ^{18}O assays, the accuracy was determined to be 0.18±2.61 o/oo.[3] The isotopic results were normalized against two international water standards: Vienna-Standard Mean Ocean Water and Standard Light Antarctic Precipitation.[4] The isotope dilution space for ^{18}O (NO) was calculated as follows:

NO (mol) = (d x A x Ea)/(a x Ed x 18.02)

where "d" was the dose of isotope water in grams; "A" was the amount of laboratory water in grams used to prepare the dose dilution; "a" was the amount of the isotope water in grams

added to the laboratory water in the dose dilution; “Ea“ was the rise in ^{18}O abundance in the laboratory water after the addition of the isotopic water; and “Ed“ is the rise in ^{18}O abundance in the urine samples at time zero obtained from the zero-time intercepts of the ^{18}O decay curves in the urine samples. The use of dose dilution in the calculation of isotope dilution spaces was recommended by the International Dietary Energy Consultancy Groups to assure accuracy of the isotope dilution calculations.[5]

TBW was calculated after correcting for 0.7% isotope sequestration for ^{18}O as follows:

TBW, moles = (NO x 103/18.02)/1.007

Fat-free mass (FFM) of the premature infants was calculated from TBW as follows:

FFM (g) = TBW x 18.02/ HC

where HC is the hydration constant of FFM based on the gestational age of each infant using the following relationship according to the body composition of the reference fetus published by Ziegler et al.[6]

HC = -0.0492 x Age (d) + 97.065

Fat mass (FM) is simply the difference between body weight and FFM.

Statistical Analysis

Weighted least squares regression modeling was used to correct the change in body weight z-score normalized to the start of the study for influencing factors (eTable 1).

$$y_{pt} = R_{pt} + T_t + T_t^2 + A_p + S_p + C_p + W_{pt} + N_p + T_t * S_p + T_t^2 * S_p + A_p * N_p + e_{pt}$$

where y_{pt} is the change in body weight z-score of the *p*th patient at the *t*th time point, R_{pt} is the random effect of the *p*th patient at the *t*th time point, T_t is the fixed effect of time at the *t*th time point, T_t^2 is the fixed effect of quadratic time at the *t*th time point, A_p is the fixed effect of the

gestational age of the *p*th patient being less than 28 at the start of the study, S_p is the fixed effect of sex of the *p*th patient, C_p is the fixed effect of the average total calories consumed of the *p*th patient, W_{pt} is the fixed effect of weekly average Na intake of the *p*th patient at the *t*th time point, N_p is the fixed effect of the sodium intervention of the *p*th patient, $T_t * S_p$ and $T_t^2 * S_p$ are the fixed effect of the interaction between linear and quadratic time and the sex of the *p*th patient, $A_p * N_p$ is the fixed effect of the interaction between the sodium intervention and the gestational age of the *p*th patient, and e_{pt} is a residual term. The model was refit with weights of the inverse variance of the model residuals to account for heteroscedasticity of the patient subgroups. Studentized model residuals passed normality checks for goodness of fit. Testing of predictor variables was done using Type II Wald X-square test. Note, a predictor variable for the average total protein consumed for each patient was tested during model construction; however, average total protein was not retained in the model because it did not significantly contribute to explaining data variation ($p_{protein} = 0.62$ vs $p_{calories} < 0.001$). Modelling and figure creation was performed in R 4.4.2 using the lme4 and lmerTest package.

To correct outcomes from the double labelled water experiment for covariates, such as FFM (1484.1 g), FM (208.4 g), body length (height, 40.5 cm), chronological age since birth (4 weeks), and weekly average sodium intake (in Study Algorithm group 5.51 mEq/kg/day), a generalized linear model was fit to the data. Estimated least squares means were extracted for the sodium intervention and sex variables and were tested for sodium intervention and sex effects using a Two-way ANOVA with Šídák's multiple comparisons test. Modelling was performed in R 4.0.2 using the lme4 and lsmeans packages, and figures were created in GraphPad Prism 10.3.0.

Supplement References

1. Schoeller DA. Energy expenditure from doubly labeled water: some fundamental considerations in humans. Am J Clin Nutr. 1983;38(6):999-1005.
2. Ellis, K. J. Human body composition: in vivo methods. Physiol Rev. 2000;80(2):649-680.
3. Wong WW, Clarke LL. Accuracy of delta$^{(18)}$O isotope ratio measurements on the same sample by continuous-flow isotope-ratio mass spectrometry. Rapid Commun Mass Spectrom. 2015;29(23):2252-2256.
4. Gonfiantini R. Standards for stable isotope measurements in natural compounds. Nature. 1978;271:534-536.
5. Prentice, A. M., International Atomic Energy Agency, Vienna (Austria), & International Dietary Energy Consultancy Group (IDECG), Lausanne (Switzerland). The doubly-labelled water method for measuring energy expenditure. Technical recommendations for use in humans. 1990
6. Ziegler EE, O'Donnell AM, Nelson SE, Fomon SJ. Body composition of the reference fetus. Growth. 1976;40(4):329-341.

eTable 1. Change in body weight (kg) z-score weighted least squares regression model details and coefficient testing.

Predictors	*Estimates*	*95% CI*	*p-value*
(Intercept)	0.618	0.30 – 0.94	**<0.001**
Group [Study Algorithm]	0.113	0.03 – 0.19	**0.007**
GA [<28]	-0.098	-0.19 - -0.01	**0.030**
Sex [M]	-0.004	-0.08 – 0.07	0.923
Time	0.034	-0.00 - -0.07	0.078
$Time^2$	-0.005	-0.01 - -0.00	**0.019**
Weekly Average Na Intake (mEq/kg/day)	-0.005	-0.02 – 0.01	0.594
Average Total Calorie Intake (kcal)	-0.005	-0.01 - -0.00	**<0.001**
Time x Sex	0.058	0.00 – 0.12	**0.047**
$Time^2$ x Sex	-0.005	-0.01 – 0.00	0.172
Group [Study Algorithm] x GA [<28]	-0.023	-0.15 – 0.11	0.725
**Random Effects**			
σ^2	2.23		
$\tau_{00\ Patient}$	0.01		
$\tau_{00\ Patient\ x\ Time}$	0.01		
$\rho_{01\ Patient}$	0.63		
ICC	0.05		
$N_{Patient}$	83 (Control ≥28 n=25, Control <28 n=18, Algorithm ≥28 n=26, Algorithm <28 n=14)		
Observations	557		
Marginal R^2	0.007		
Conditional R^2	0.053		
Asymptotic one-sample Kolmogorov-Smirnov test on studentized residuals	0.052		0.10

eTable 2. Number of babies who received human milk and formula by week

Week		Control (N=43)	Intervention (N=43)	Total (N=86)	p value
1	Human milk, n (%)	43 (100.0%)	42 (97.7%)	85 (98.8%)	1.000[1]
	Formula, n (%)	2 (4.7%)	0 (0.0%)	2 (2.3%)	0.494[1]
2	Human milk, n (%)	43 (100.0%)	42 (97.7%)	85 (98.8%)	1.000[1]
	Formula, n (%)	1 (2.3%)	0 (0.0%)	1 (1.2%)	1.000[1]
3	Human milk, n (%)	43 (100.0%)	42 (97.7%)	85 (98.8%)	1.000[1]
	Formula, n (%)	4 (9.3%)	1 (2.3%)	5 (5.8%)	0.360[1]
4	Human milk, n (%)	40 (93.0%)	39 (90.7%)	79 (91.9%)	1.000[1]
	Formula, n (%)	8 (18.6%)	8 (18.6%)	16 (18.6%)	1.000[1]
5	Human milk, n (%)	37 (86.0%)	32 (74.4%)	69 (80.2%)	0.279[1]
	Formula, n (%)	12 (27.9%)	14 (32.6%)	26 (30.2%)	0.815[1]
6	Human milk, n (%)	30 (71.4%)	27 (64.3%)	57 (67.9%)	0.641[1]
	Formula, n (%)	16 (38.1%)	18 (42.9%)	34 (40.5%)	0.824[1]
7	Human milk, n (%)	23 (54.8%)	24 (58.5%)	47 (56.6%)	0.826[1]
	Formula, n (%)	15 (35.7%)	15 (36.6%)	30 (36.1%)	1.000[1]
8	Human milk, n (%)	19 (47.5%)	20 (48.8%)	39 (48.1%)	1.000[1]
	Formula, n (%)	13 (32.5%)	16 (39.0%)	29 (35.8%)	0.645[1]

[1]Fisher's Exact Test for Count Data
